# *Aconitum pseudo-laeve var. erectum* Inhibits Receptor Activator of Nuclear Factor Kappa-B Ligand-Induced Osteoclastogenesis via the c-Fos/nuclear Factor of Activated T-Cells, Cytoplasmic 1 Signaling Pathway and Prevents Lipopolysaccharide-Induced Bone Loss in Mice

**DOI:** 10.3390/molecules190811628

**Published:** 2014-08-05

**Authors:** Jong Min Baek, Ju-Young Kim, Yoon-Hee Cheon, Sun-Hyang Park, Sung-Jun Ahn, Kwon-Ha Yoon, Jaemin Oh, Myeung Su Lee

**Affiliations:** 1 Department of Anatomy, School of Medicine, Wonkwang University, Iksan, Jeonbuk 570-749, Korea; E-Mails: phone8418@hanmail.net (J.M.B.); hanleuni@naver.com (Y.-H.C.); beryls@wku.ac.kr (S.-H.P.); asj0427@naver.com (S.-J.A.); 2 BK21plus Program & Department of Smart Life-Care Convergence, Graduate School, Wonkwang University, Iksan, Jeonbuk 570-749, Korea; 3 Imaging Science-based Lung and Bone Diseases Research Center, Wonkwang University, Iksan, Jeonbuk 570-749, Korea; E-Mails: kimjy1014@gmail.com (J.-Y.K.); khy1646@wku.ac.kr (K.-H.Y.); 4 Department of Radiology, School of Medicine, Wonkwang University, Iksan, Jeonbuk 570-749, Korea; 5 Institute for Skeletal Disease, Wonkwang University, Iksan, Jeonbuk 570-749, Korea; 6 Division of Rheumatology, Department of Internal Medicine, Wonkwang University, Iksan, Jeonbuk 570-749, Korea

**Keywords:** *Aconitum pseudo-laeve var. erectum*, osteoclast, bone, osteoporosis

## Abstract

*Aconitum pseudo-laeve var. erectum* (APE) has been widely shown in herbal medicine to have a therapeutic effect on inflammatory conditions. However, there has been no evidence on whether the extract of APE is involved in the biological bone metabolism process, particularly osteoclast-mediated bone resorption. In this study, we confirmed that the administration of APE could restore normal skeletal conditions in a murine model of lipopolysaccharide (LPS)-induced bone loss via a decrease in the receptor activator of nuclear factor kappa-B ligand (RANKL)/osteoprotegerin (OPG) ratio and osteoclast number. We then investigated the effect of APE on the RANKL-induced formation and function of osteoclasts to elucidate its underlying molecular mechanisms. APE suppressed the formation of tartrate-resistant acid phosphatase (TRAP)-positive cells, as well as the bone-resorbing activity of mature osteoclasts. Furthermore, APE attenuated nuclear factor of activated T-cells, cytoplasmic 1 (NFATc1) and c-Fos without affecting any early signal pathway of osteoclastogenesis. Subsequently, APE significantly downregulated the expression of various genes exclusively expressed in osteoclasts. These results demonstrate that APE restores LPS-induced bone loss through a decrease of the serum RANKL/OPG ratio, and inhibits osteoclast differentiation and function, suggesting the promise of APE as a potential cure for various osteoclast-associated bone diseases.

## 1. Introduction

With a rapidly aging society, metabolic bone disorders including osteoporosis, periodontitis, Paget’s disease, and rheumatoid arthritis have become major health problems [1]. Of these, osteoporosis, leading to the deterioration of skeletal condition, is directly associated with excessive osteoclast bone resorption activity and is particularly common in postmenopausal women [2,3]. Therefore, various studies have been undertaken to identify novel therapeutic agents for the treatment of osteoporosis, targeting the functions of two main specific types of bone cells: resorption of old bone by osteoclasts and formation of new bone by osteoblasts.

To modulate the differentiation of multinucleated bone resorbing osteoclasts, the induction of several early signaling cascades, which are dependent on the receptor activator of nuclear factor kappa-B ligand (RANKL)-RANK axis, is required [4,5,6]. By activating these signaling pathways—which consist of mitogen activated protein kinases (MAPKs) comprising p38, c-Jun N-terminal kinase (JNK), and extracellular signal-regulated kinase (ERK), as well as NF-κB, Akt, and phospholipase Cγ2 (PLCγ2)—both c-Fos and nuclear factor of activated T-cells, cytoplasmic 1 (NFATc1), as two main transcription factors of osteoclastogenesis, translocate into the nucleus of osteoclast precursors [7,8,9,10]. Thereafter, the expression of osteoclast-specific marker genes—including osteoclast-associated receptor (OSCAR), tartrate-resistant acid phosphatase (TRAP), Atp6v0d2, cathepsin K, as well as, osteoclast stimulatory transmembrane protein (OC-STAMP), dendritic cell-specific transmembrane protein (DC-STAMP), calcitonin receptor (CTR), and matrix metallopeptidase 9 (MMP-9)—is finally induced to develop characteristic osteoclasts via interaction between c-Fos and NFATc1 [10,11,12,13,14,15]. Previously, both c-Fos and NFATc1 were proven to have a critical role in osteoclast formation and differentiation. c-Fos, which is a component of the transcription protein complex AP-1 cooperates with NFATc1—a member of the transcriptional NFAT family—by binding to the promoter region of specific genes in order to regulate osteoclastogenesis. It has been demonstrated that c-Fos^−/−^ mice display the osteopetrotic phenomenon, owing to the impairment of osteoclastic activity [16]. Moreover, the ectopic expression of NFATc1 efficiently induces bone marrow macrophages (BMMs) to differentiate into mature osteoclasts despite of the absence of RANKL [10]. Because of the pivotal role of these transcription factors, it is important to regulate the activation of both c-Fos and NFATc1 in the treatment of bone-related diseases, such as osteoporosis.

Based on previous research, which identified the potential value of natural plants in the prevention of osteoporosis [17,18], we selected the ethanol extract of *Aconitum pseudo-laeve var. erectum* (APE) using analysis with TRAP staining. Previously, APE has been demonstrated to have pharmacological effects on blood circulation and inflammation [19,20]. However, there is no evidence that it has any therapeutic effects in relation to bone metabolism. In the present study, we demonstrated the inhibitory effect and mechanism of action of APE on RANKL-induced osteoclastogenesis, as well as the *in vivo* effect of APE in a murine model of lipopolysaccharide (LPS)-induced bone erosion.

## 2. Results and Discussion

### 2.1. Administration of APE Restores LPS-Induced Bone Loss in Vivo

**Figure 1:**
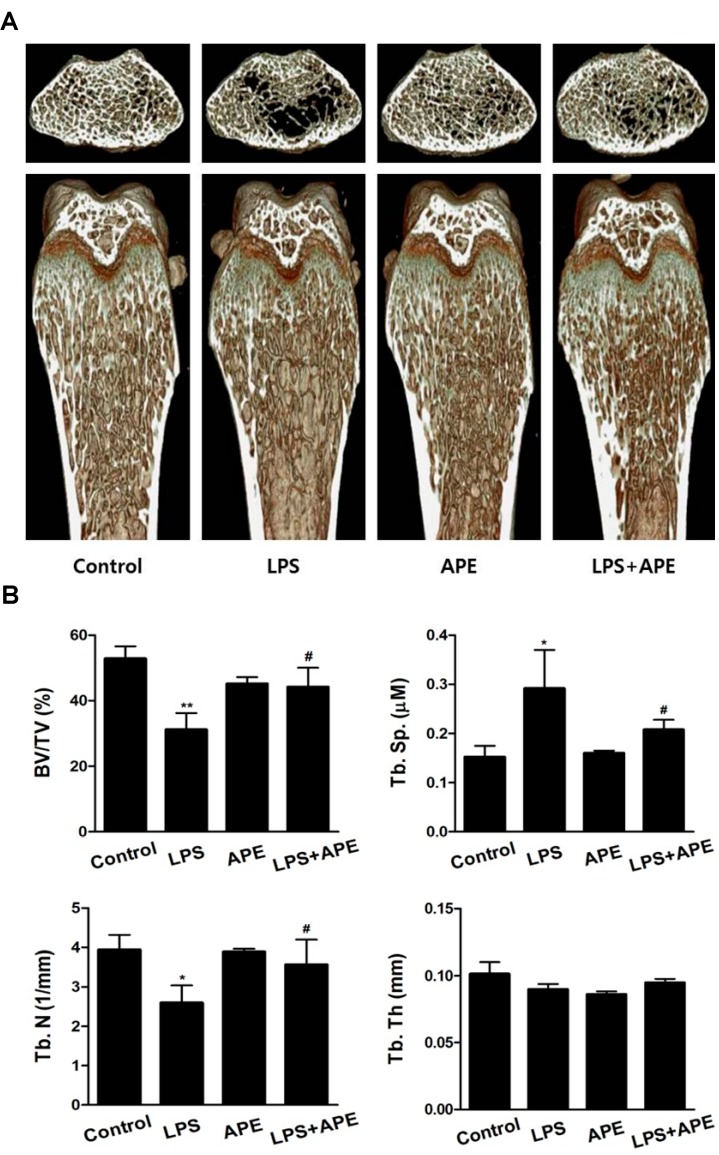
*Aconitum pseudo-laeve var. erectum* (APE) regulates lipopolysaccharide (LPS)-induced bone erosion in a mouse model. (**A**) Mice were sacrificed 8 days after the first LPS injection and radiographs of the longitudinal and transverse section of the proximal femurs were obtained using a micro-computed tomography (micro-CT) apparatus; (**B**) The bone volume per tissue volume (BV/TV), trabecular separation (Tb.Sp), Tb.Th, and trabecular number (Tb.N) of the femurs were determined using the micro-CT data as analyzed with INFINITT-Xelis software.

To determine the effect of APE on bone mass *in vivo*, we examined the effect of APE in murine models of LPS-mediated bone erosion. It was reported that injection of LPS, which regulates inflammatory responses in the immune system, induced an increase in osteoclast surface and number, as well as excessive osteoclastic bone resorption, resulting in a loss of bone density *in vivo* [21,22]. In accordance with previous studies, mice were intraperitoneally injected with PBS as a control and LPS as a bone erosion model. After 8 days, the left femurs of sacrificed mice were used for micro-computerized tomography (micro-CT) analysis. Although a greater reduction of bone mass was observed in the femurs of LPS-injected mice compared to the vehicle-treated mice, a partial recovery of bone density of both LPS- and APE-treated mice was observed in 3-dimensional visualization (Figure 1A). Morphometric analysis of the femurs of LPS-injected mice showed a decrease of bone volume per tissue volume (BV/TV) and trabecular number (Tb.N), and an increase of trabecular separation (Tb.Sp). We observed that the reduction of BV/TV, Tb.N, and Tb.Sp following LPS injection was recovered in the APE-treated, LPS-induced mice (Figure 1B). These results indicate that APE inhibits inflammatory-induced bone loss *in vivo* (Figure 1). Although oral administration of APE could have anti-osteoprotic effect on the short-term LPS-induced bone loss, it is still considered to investigate the possibility of APE has recovery effect on other bone loss mice model during long-term. Thus, our further investigation is needed to clarify the effects of APE in other long-term *in vivo* bone loss model, such as ovariectomized (OVX) or immobilized model, except the short-term LPS-induced bone loss model. 

### 2.2. APE Restores LPS-Induced Bone Loss by Inhibiting the RANKL/OPG Ratio and Osteoclast Formation

**Figure 2:**
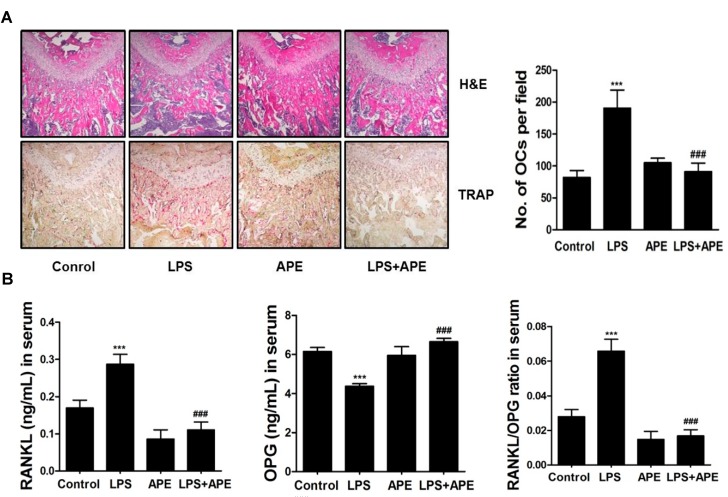
(**A**) Dissected femora were fixed, decalcified, embedded, and sectioned. Sections were stained with hematoxylin and eosin (H&E) (top) and with tartrate-resistant acid phosphatase (TRAP) (bottom). The number of osteoclasts per field of tissue was analyzed using the histomorphometric results (right); (**B**) Levels of receptor activator of nuclear factor kappa-B ligand (RANKL), osteoprotegerin (OPG), and the serum RANKL/OPG ratio of control, LPS-treated, *Aconitum pseudo-laeve var. erectum* (APE)-treated, and both LPS and APE-treated mice.

To investigate the mechanism of the inhibitory effects of APE on LPS-induced bone loss, we examined the effect of APE on the RANKL/OPG ratio and osteoclast formation. As shown in Figure 2A, APE reduced bone erosion and osteoclast formation in the trabecular bone region. As expected, the number of osteoclasts located in the femur was significantly suppressed in LPS and APE-treated mice. Furthermore, to confirm whether the APE alteration of RANKL/OPG production contributed to this effect, we performed an enzyme-linked immunosorbent assay (ELISA) using the serum of mice in the experimental group. Surprisingly, APE reduced the increase in the levels of RANKL and ameliorated the decrease in the level of OPG in LPS-treated mice. 

*: *p* < 0.05; **: *p* < 0.01 *versus* control; ^#^: *p* < 0.05 *versus* lipopolysaccharide (LPS) group.

As anticipated, the ratio of RANKL/OPG was significantly reduced in response to treatment with APE (Figure 2B). RANKL is a transmembrane protein expressed in osteoblast and stromal cells, playing a role as an initiator of osteoclast differentiation by binding to its receptor, RANK [6,23]. OPG, which is considered a decoy receptor of RANKL, is generally expressed in tissues, including the heart, kidney, and bone marrow and inhibits the differentiation of osteoclast precursors by binding to RANKL [6]. Previous reports have verified that an increase in the RANKL/OPG ratio is the main cause for enhancement of osteoclast activity, leading to excessive bone resorption and subsequent low density of bone mass [24,25]. In summary, APE partially recovers bone destruction through an increase in osteoclast formation in the region of trabecular architecture by modulating the RANKL/OPG ratio, down-regulating RANKL, and up-regulating OPG expression (Figure 2).

***: *p* < 0.001 versus control; ^###^: *p* < 0.001 versus lipopolysaccharide (LPS) group.

Additionally, we calculated the direct effect of APE on RANKL and OPG mRNA expression in bone cells, primary osteoblasts, *in vitro*. In results, APE did not directly affect mRNA expression of both RANKL and OPG induced by IL-1 (Supplementary Figure S1). In Figure 2B, on the contrary, APE altered the serum levels of RANKL and OPG in the condition of LPS stimulation. Previously, it is proved that circulating soluble RANKL acts as an indicator of bone turnover marker. Also, this circulating form of RANKL contains two forms of RANKL, a truncated ectodomain cleaved from the cell-bound form and a primary secreted form produced by activated T cells. However, trimeric transmembrane protein secreted from osteoblasts is not comprised in circulating RANKL [26]. Taken together, we assumed that APE only affected the expression of circulating soluble form of RANKL apart from the form of molecule derived from osteoblast, suggesting that the recovery effect of APE on LPS-induced bone loss is caused by blocking two forms of circulating RANKL.

### 2.3. APE Prevents the Formation of TRAP-positive Osteoclasts and the Bone Resorbing Ability of Mature Osteoclasts

**Figure 3:**
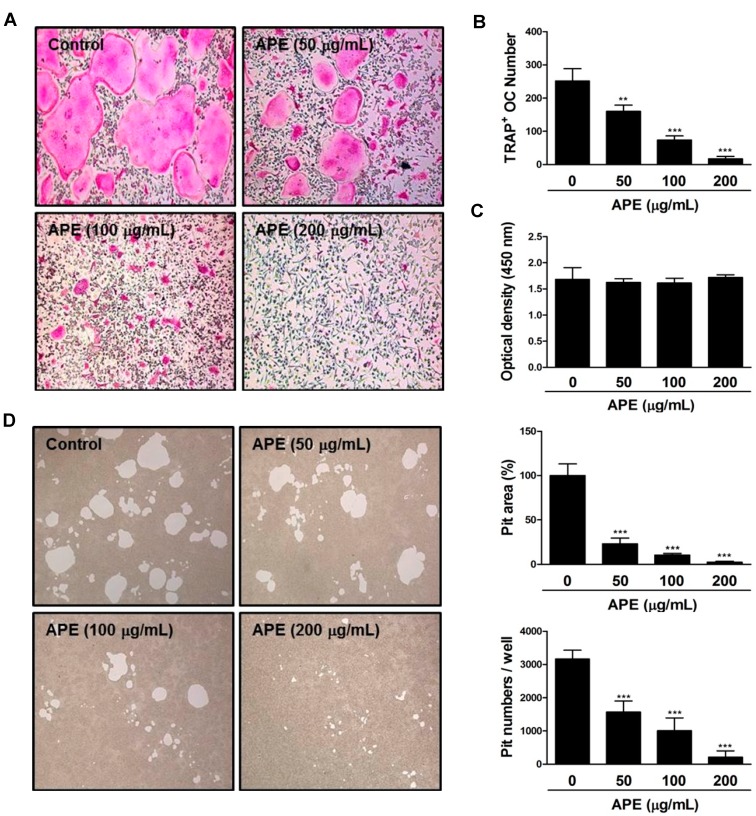
*Aconitum pseudo-laeve var. erectum* (APE) inhibits the formation of tartrate-resistant acid phosphatase (TRAP)-positive osteoclasts, as well as the bone resorbing activity of mature osteoclasts. (**A**) Bone marrow macrophages (BMMs) were cultured for 4 days in the presence of macrophage colony-stimulating factor (M-CSF; 30 ng/mL) and receptor activator of nuclear factor kappa-B ligand (RANKL; 100 ng/mL) with dimethyl sulfoxide (DMSO; control) or APE. Cells were fixed with 3.7% formalin in phosphate-buffered saline (PBS), permeabilized with 0.1% Triton X-100 in PBS and stained with TRAP solution; (**B**) TRAP-positive multinucleated cells were counted as osteoclasts; (**C**) BMMs were cultured for 3 days at the indicated doses of APE in the presence of M-CSF (30 ng/mL). Cell viability was analyzed by XTT assay; (**D**) Mature osteoclasts were seeded on hydroxyapatite-coated plates for 24 h with the indicated concentrations of APE. Attached cells on the plates were removed and photographed under a light microscope. The relative ratio and number of pit areas was quantified using Image J.

To investigate the effect of APE on osteoclast formation and function *in vitro*, we initially cultured primary BMMs treated with several concentrations of APE or dimethyl sulfoxide (DMSO; as a control). It is well known that hematopoietic precursor cells of the macrophage/monocyte lineage differentiate into multinucleated osteoclasts in the presence of macrophage colony-stimulating factor (M-CSF) and RANKL [27,28]. In the process of differentiation, the expression of TRAP, which is deeply associated with various stages of the skeletal system—such as collagen synthesis—is remarkably increased [29]. The formation of TRAP-positive multinuclear cells (MNCs) appeared distinctly in BMMs treated with DMSO. However, BMMs treated with APE were limited to differentiate into osteoclasts in a dose-dependent manner (Figure 3A). Furthermore, the number of TRAP-positive osteoclasts was dramatically decreased (Figure 3B). Subsequently, we analyzed an XTT assay to explore the possibility that the inhibitory effect of APE on osteoclastogenesis is associated with cytotoxicity. At the indicated concentrations, APE did not affect cell viability, even at a high concentration (Figure 3C). These results indicate that the formation of TRAP-positive multinucleated giant cells is significantly suppressed by APE treatment, with no cytotoxicity. In the development of mature osteoclasts, bone-resorbing activity is in charge of main function of it. The formation of a sealing zone and ruffled border occurs in order to degrade the extracellular bone matrix [30,31,32]. The sealing zone, which contains abundant actin filaments (f-actin), plays a key role in attachment to the bone surface through the expression of integrin αν and β_3_ [33,34]. Simultaneously, the formation of a ruffled border occurs in the middle of the sealing zone. To dissolve the bone matrix, the ruffled border modifies the acidic environment through the secretion of proton ions and lysosomal proteases [35,36]. In an effort to identify the effect of APE on bone resorbing activity, we seeded mature osteoclasts on top of a hydroxyapatite-coated plate with or without APE. Although the formation of a considerable resorption pit was observed in DMSO conditions, there was a dose-dependent suppression of the formation of resorption pits on the plates by mature osteoclasts treated with APE. Furthermore, the relative ratio and number of pit areas were decreased in a dose-dependent manner (Figure 3D). These results suggest that APE has a suppressive effect on the bone matrix-dissolving activity of mature osteoclasts (Figure 3).

**: *p* < 0.01; ***: *p* < 0.001.

### 2.4. The Inhibitory Effect of APE on c-Fos and NFATc1 Activation Is Not Associated with Early Signal Pathways

**Figure 4:**
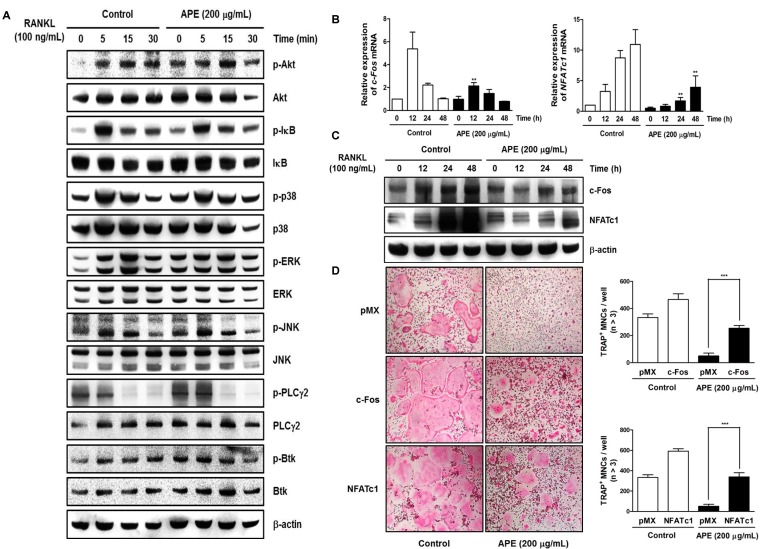
*Aconitum pseudo-laeve var. erectum* (APE) suppresses receptor activator of nuclear factor kappa-B ligand (RANKL)-induced c-Fos and nuclear factor of activated T-cells, cytoplasmic 1 (NFATc1) expression without stimulating early signal pathways. (**A**) Bone marrow macrophages (BMMs) were pretreated with DMSO (control) or APE (200 µg/mL) for 1 h in the presence of macrophage colony-stimulating factor (M-CSF; 30 ng/mL) and were stimulated with RANKL (100 ng/mL) for the indicated times. Whole-cell lysates underwent western blot analysis with the various indicated antibodies. β-actin served as the internal control; (**B**) BMMs were stimulated with RANKL (100 ng/mL) and M-CSF (30 ng/mL) in the presence or absence of APE (200 µg/mL) for the indicated times. Total RNA was isolated from cells using QIAzol reagent and mRNA expression levels of c-Fos and NFATc1 were evaluated using real-time PCR; (**C**) Effects of APE on protein expression levels of c-Fos and NFATc1 were evaluated using western blot analysis. β-actin was used as the internal control; (**D**) BMMs were infected with retroviruses expressing pMX-IRES-EGFP (pMX), pMX-NFATc1-EGFP, and pMX-cFos-EGFP. Infected BMMs were cultured with or without APE (200 µg/mL) in the presence of M-CSF (30 ng/mL) and RANKL (100 ng/mL) for 4 days. After culturing, the cells were fixed and stained for tartrate-resistant acid phosphatase (TRAP) (left). The TRAP-positive multinucleated osteoclasts were counted (right).

To figure out whether APE stimulates the phosphorylation of early signaling, we tested the effect of APE on MAP kinases—including p38, ERK, JNK, and Akt, IκB—as well as calcium signaling pathways, such as PLCγ2, and Bruton's tyrosine kinase (BTK). Surprisingly, APE did not affect any early signaling pathway of RANKL-induced osteoclastogenesis (Figure 4A). Thus, we suggest that APE could regulate the differentiation of osteoclasts via directly targeting c-Fos/NFATc1 signaling. Previous studies revealed that NFATc1 is a downstream target gene of c-Fos in the late stage of osteoclastogenesis. 

Osteoclast precursor cells, which are deficient in c-Fos, showed a low expression of NFATc1 activation and recovered normal osteoclast formation and bone resorption ability, as well as transcription activity of osteoclast-specific genes through the inducement of the NFATc1 active form [37,38]. To demonstrate the effect of APE on c-Fos/NFATc1 signaling, we examined the effect of APE on the protein levels of c-Fos and NFATc1 by western blotting, and the mRNA levels of the two transcription factors through real-time PCR.

c-Fos and NFATc1 were suppressed significantly in comparison with the control (Figure 4B,C). Additionally, to confirm whether c-Fos and NFATc1 expression is sufficient to reverse the effect of APE on osteoclastogenesis, we applied a retrovirus to overexpress c-Fos and NFATc1 (Supplementary Figure S2). As shown in Figure 4D, the ectopic expression of c-Fos and NFATc1 recovered the inhibitory effect of APE on osteoclast differentiation. These results show that APE blocks the activation of both c-Fos and NFATc1 without affecting the RANKL-dependent signaling pathway (Figure 4).

### 2.5. APE Regulates Osteoclastogenesis through Suppressing the mRNA Expression of Osteoclast Marker Genes 

**Figure 5:**
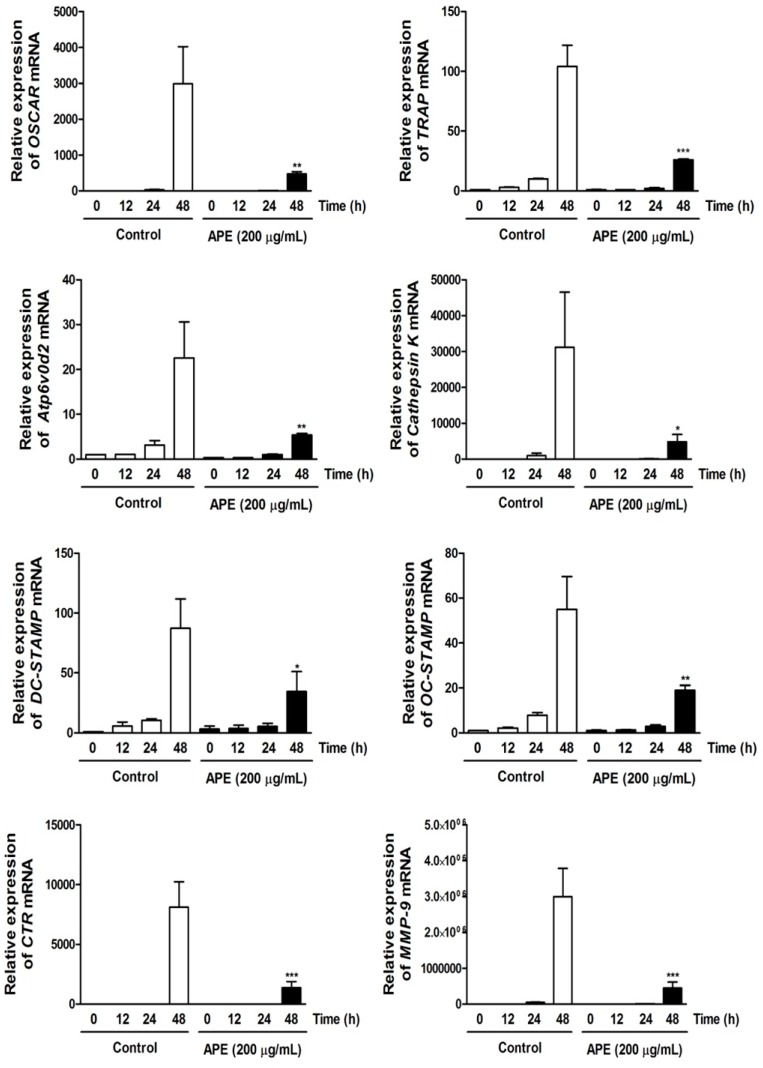
*Aconitum pseudo-laeve var. erectum* (APE) down-regulates the expression of osteoclast-specific marker genes. Bone marrow macrophages (BMMs) were stimulated with receptor activator of nuclear factor kappa-B ligand (RANKL; 100 ng/mL) and macrophage colony-stimulating factor (M-CSF; 30 ng/mL) in the presence or absence of APE (200 µg/mL) for the indicated times. Total RNA was isolated from cells using QIAzol reagent and mRNA expression levels of osteoclast-associated receptor (OSCAR), tartrate-resistant acid phosphatase (TRAP), Atp6v0d2, Cathepsin K, dendritic cell-specific transmembrane protein (DC-STAMP), osteoclast stimulatory transmembrane protein (OC-STAMP), calcitonin receptor, and matrix metallopeptidase 9 (MMP-9) were evaluated by real-time PCR.

We analyzed the effect of APE on the expression of various osteoclast-specific genes that results from the transcriptional activity of NFATc1. APE significantly downregulated the transcription of OSCAR, TRAP, DC-STAMP, OC-STAMP, Atp6v0d2, CTR, and MMP-9 (Figure 5). In particular, the expression of Cathepsin K—the main enzyme for cleaving extracellular matrix—was restricted in response to APE treatment (Figure 5). Previously, it was revealed that Cathepsin K-deficient mice underwent osteopetrosis due to impairment of bone resorption. Further, the lack of protein kinase-C-delta (PKCδ), which regulates Cathepsin K exocytosis by stimulating actin cross-linking protein myristoylated alanine-rich C-kinase substrate (MARCKS), shows preventive effects on postmenopausal osteoporosis [39,40]. These results indicate that APE negatively stimulates osteoclast differentiation and bone resorbing activity via regulating the various marker genes (Figure 5).

*: *p* < 0.05; **: *p* < 0.01; ***: *p* < 0.001.

*: *p* < 0.05; **: *p* < 0.01; ***: *p* <0.001.

## 3. Experimental Section 

### 3.1. Reagents and Antibodies

The 95% ethanol extract of APE was purchased from the Korean Plant Extract Bank (Daejeon, Korea). TRAP staining solution was from Sigma Aldrich (St. Louis, MO, USA), the XTT assay kit was obtained from Roche (Indianapolis, IN, USA). The α-minimum essential medium (α-MEM), fetal bovine serum (FBS), and penicillin-streptomycin were purchased from Gibco-BRL (Grand Island, NY, USA) and soluble human recombinant M-CSF and RANKL were purchased from Peprotech (London, UK). Specific antibodies against phospho-PLCγ2 (sc-101785), PLCγ2 (sc-5283), IκB (sc-371), c-Fos (sc-7202), and NFATc1 (sc-7294) were obtained from Santa Cruz Biotechnology (Santa Cruz, CA, USA) and phospho-Btk (GTX61791) was purchased from GeneTex (Irvine, CA, USA). Specific primary antibodies against phospho-p38 (#9211), p38 (#9212), phospho-Akt (#9271), Akt (#9272), phospho-JNK (#9251), JNK (#9252), phospho-IκB (#2859), phospho-ERK (#9101), ERK (#9102), and Btk (#3533) were purchased from Cell Signaling Technology (Beverly, MA, USA) and β-actin (A5441; housekeeping gene) was obtained from Sigma Aldrich.

### 3.2. Mouse Bone Marrow Cell (BMC) Isolation and Osteoclast Differentiation

Mouse BMCs were obtained from the femurs and tibiae of a 5-week-old ICR mouse and were incubated in α-MEM with 10% FBS, 1% penicillin/streptomycin, M-CSF (10 ng/mL) for 1 day to obtain non-adherent cells. The non-adherent cells as osteoclast precursors were incubated in α-MEM with 10% FBS, 1% penicillin/streptomycin, M-CSF (30 ng/mL) for 3 days. After 3 days, the adherent cells were used as BMMs. The BMMs were incubated with M-CSF (30 ng/mL) and RANKL (100 ng/mL) in the presence of APE (50–200 µg/mL) or DMSO as control. After 3 days, the culture media was changed under the same conditions. After 1 day, the cells were fixed in 3.7% formalin for 20 min and permeabilized with 0.1% Triton X-100 and then stained with TRAP solution, and the stained cells were counted to establish the level of osteoclast differentiation. 

### 3.3. Cell Viability Assay

The BMMs (1 × 10^4^ cells/well) were cultured with or without APE (50–200 µg/mL) for 3 days in the presence of M-CSF (30 ng/mL) in 96-well plates. After 4 h incubation of the cells in a medium containing 50 µL of XTT solution (sodium 3'-[1-(phenyl-aminocarbonyl)-3,4-tetrazolium]-bis(4-methoxy-6-nitro), benzenesulfonic acid hydrate, and N-methyl dibenzopyrazine methyl sulfate), the optical density was measured as 450 nm using an ELISA reader (Molecular Devices, Sunnyvale, CA, USA).

### 3.4. Western Blot Analysis 

The BMMs were lysed in a lysis buffer containing 50 mM Tris-HCl, 150 mM NaCl, 5 mM ethylenediaminetetraacetic acid (EDTA), 1% Triton X-100, 1 mM sodium fluoride, 1 mM sodium vanadate, 1% deoxycholate, protease inhibitors and the lysate was centrifuged at 14,000 rpm for 20 min to obtain pure protein. The protein concentration was measured using a Bio-Rad colorimetric protein assay kit (Bio-Rad Laboratories Inc., Hercules, CA, USA) and equal amounts of proteins were separated through an SDS-polyacrylamide gel. The proteins were transferred to a polyvinylidene difluoride (PVDF) membrane (Millipore, Bedford, MA, USA) and treated with 5% non-fat dry milk to inhibit attachment of non-specific proteins. After the membrane was treated with primary and secondary antibodies (horseradish peroxidase [HRP]-conjugated sheep anti-mouse or donkey anti-rabbit immunoglobulin), the expression of specific protein signals was measured using a chemiluminescence detection system (Millipore).

### 3.5. Quantitative Real-time PCR Analysis

Total RNA was extracted using QIAzol lysis reagent (Qiagen, Valencia, CA, USA) according to the manufacturer’s instructions and equal amounts of the cDNA of RNA were synthesized from 1 µg of total RNA using SuperScript II Reverse Transcriptase (Invitrogen, San Diego, CA, USA). Real-time PCR was performed using a Exicycler™ 96 Real-Time Quantitative Thermal Block (Bioneer Co., Daejeon, Korea) in a 20 µL reaction mixture containing 10 µL SYBR Green Premix (Bioneer Co.), 10 pmol forward primer, 10 pmol reverse primer, and 1 µg cDNA. The detection program of real-time PCR proceeded according to the following protocols: initial denaturation at 95 °C for 5 min and 40 cycles of 3 steps PCR (denaturation at 95 °C for 1 min, annealing at 60 °C for 30 s, and extension at 72 °C for 1 min). Gene expression levels were normalized to the housekeeping gene, β-actin.

The relative results of specific genes were calculated using the comparative cycle threshold method. Table 1 shows the primer sets used in the Real-time PCR.

**Table 1:** Primer sequences used for real-time PCR analysis.

Primer sequences for Real-time RT-PCR	
c-Fos	Forward: 5'-GGT GAA GAC CGT GTC AGG AG-3'
	Reverse: 5'-TAT TCC GTT CCC TTC GGA TT-3'
NFATc1	Forward: 5'-GAG TAC ACC TTC CAG CAC CTT-3'
	Reverse: 5'-TAT GAT GTC GGG GAA AGA GA-3'
TRAP	Forward: 5'-ACT TCC CCA GCC CTT ACT AC-3'
	Reverse: 5'-TCA GCA CAT AGC CCA CAC CG-3'
OSCAR	Forward: 5'-GGA ATG GTC CTC ATC TGC TT-3'
	Reverse: 5'-TCC AGG CAG TCT CTT CAT TTT-3'
Cathepsin K	Forward: 5'-CCA GTG GGA GCT ATG GAA GA-3'
	Reverse: 5'-CTC CAG GTT ATG GGC AGA GA -3'
Atp6v0d2	Forward: 5'-GAC CCT GTG GCA CTT TTT GT-3'
	Reverse: 5'-GTG TTT GAG CTT GGG GAG AA-3'
DC-STAMP	Forward: 5'-TCC TCC ATG AAC AAA CAG TTC CA-3'
	Reverse: 5'-AGA CGT GGT TTA GGA ATG CAG CTC-3'
OC-STAMP	Forward: 5'-ATG AGG ACC ATC AGG GCA GCC ACG-3'
	Reverse: 5'-GGA GAA GCT GGG TCA GTA GTT CGT-3'
Calcitonin receptor (CTR)	Forward: 5'-TCC AAC AAG GTG CTT GGG AA-3'
	Reverse: 5'-CTT GAA CTG CGT CCA CTG GG-3'
MMP-9	Forward: 5'-TCC AAC CTC ACG GAC ACC C-3'
	Reverse: 5'-AGC AAA GCC GGC CGT AGA-3'
GAPDH	Forward: 5'-TCA AGA AGG TGG TGA AGC AG-3'
	Reverse: 5'-GGT GGA GGA GTG GGT GTC-3'

### 3.6. Bone Resorption Assay

To obtain mature osteoclasts, BMCs and primary osteoblasts were co-cultured in collagen gel-coated culture dishes for 7 days in the presence of 1,25-dihydroxyvitamin D_3_ (Vit D_3_; Sigma) and prostaglandin E2 (PGE2; Sigma). After 7 days, the co-cultured cells were detached using 0.1% collagenase at 37 °C for 10 min and the separated cells were replated on dentin slices or hydroxyapatite-coated plates (Corning, NY, USA) with or without APE (200 µg/mL) for 24 h or 48 h. Subsequently, the cells on the plates were removed and the resorption pits were photographed and quantified using the Image-Pro Plus program version 4.0 (Media Cybernetics, Silver Spring, MD, USA).

### 3.7. Retroviral Gene Transfection

Packaging of the retroviral vectors pMX-IRES-EGFP, pMX-cFos-IRES-EGFP, and pMX-NFATc1-IRES-EGFP was performed using transient transfection of these pMX vectors into Plat-E retroviral packaging cells using X-tremeGENE 9 (Roche, Nutley, NJ, USA) according to the manufacturer’s protocol. After incubation in fresh medium for 2 days, the culture supernatants of the retrovirus-producing cells were collected. For retroviral infection, non-adherent BMCs were cultured in M-CSF (30 ng/mL) for 2 days. The BMMs were incubated with viral supernatant pMX-IRES-EGFP, pMX-cFos-IRES-EGFP, and pMX-NFATc1-IRES-EGFP virus-producing Plat-E cells together with polybrene (10 µg/mL) and M-CSF (30 ng/mL) for 6 h. The infection efficiency of the retrovirus was determined by green fluorescent protein expression and was always >80%. After infection, the BMMs were induced to differentiate in the presence of M-CSF (30 ng/mL) and RANKL (100 ng/mL) for 4 days. The forced expression of each construct and osteoclast formation were detected using a fluorescence microscope and TRAP staining.

### 3.8. Mouse Model of LPS-induced Bone Erosion and Micro-CT and Histological Analysis

Male, 5-week-old ICR mice were purchased from Damul Science (Daejeon, Korea). The mice were kept in a temperature (22 °C–24 °C) and humidity (55%–60%) controlled environment with a 12 h light/dark cycle. The use of experimental animals has been reviewed by the IACUC and has been approved under WKU14-40. The ICR mice were divided into 4 main experimental groups comprising 5 mice each: phosphate-buffered saline (PBS)-treated (Control), only LPS-treated (LPS), only APE-treated (APE), and both LPS and APE-treated (LPS+APE). APE (200 mg/kg) or PBS as a control was administered orally 1 day before LPS injection (5 mg/kg). APE or PBS was administered orally every other day for 8 d. LPS was injected intraperitoneally on days 1 and 4. The mice were sacrificed after 8 days, and the left femurs underwent high-resolution micro-CT analysis. The intact femur metaphysic regions were scanned by micro-CT (NFR-Polaris-S160; Nanofocus Ray, Iksan, Korea) with a source voltage of 60 kVp, current of 114 mA, and 7 mm isotropic resolution. Femur scans were performed from the growth plate approximately to 2 mm, with a total of 350 sections per scan. After 3D reconstruction, BV/TV, Tb.N, Tb.Th, and Tb.Sp were calculated using INFINITT-Xelis software (INFINITT Healthcare, Seoul, Korea). The right femurs were fixed in 4% neutral buffered paraformaldehyde (Sigma) for 1 day, decalcified for 3 weeks in 12% EDTA and embedded in paraffin. Sections of 5-µm thickness were prepared using a Leica microtome RM2255 (Leica Microsystems, Bannockburn, IL, USA). For histologic examination, sections were stained with hematoxylin and eosin (H&E) and another section was stained with TRAP to identify osteoclasts on the bone surface. Parameters for bone resorption—including number of osteoclasts per field of tissue—were quantified using the Image Pro-Plus program, version 4.0 (Media Cybernetics). Nomenclature, symbols, and units used in this study are those recommended by the American Society for Bone Mineral Research (ASBMR) Nomenclature Committee.

### 3.9. Measurement of RANKL and OPG

The serum levels of RANKL and OPG were determined using commercial ELISA kits (RANKL: Cat. NO. MTR00; OPG: Cat. No. MOP00) (R&D Systems, Minneapolis, MN, USA) in conformity with the following instructions. Briefly, all microtitre plates were coated overnight at 4 °C with antibodies against mouse RANKL and OPG. Subsequently, the plates were blocked, samples and standards were added at various dilutions in duplicate and incubated at 4 °C for 24 h. Plates were washed 3 times with buffer, and antibodies were added to the wells. Plates were incubated at room temperature for 1 h, washed, and 50 µL of avidin-HRP was added. The color reagent o-phenyl-enediamine was added 15 min later, and the plates were incubated in the dark at 37 °C for 15–20 minutes. The enzyme reaction was stopped with H_2_SO_4_ and the absorbance was measured at 490 nm. Values were expressed in pg/mL.

### 3.10. Statistical Analysis

Experiments were conducted separately at least three times and all data are presented as the mean ± standard deviation (SD). All statistical analyses were performed by using SPSS (Korean version 14.0). The statistical differences were analyzed using one-way analysis of variance (ANOVA) followed by Tukey’s *post hoc* test. *p*-values less than 0.05 were considered significant.

## 4. Conclusions 

APE restored LPS-induced bone erosion in a murine model and it was revealed that its underlying mechanism was APE inhibition of osteoclast differentiation and bone resorbing activity through direct inactivation of both c-Fos and NFATc1, without affecting various RANKL-induced early signaling pathways, such as MAPKs and calcium signaling. Consequently, it is the first demonstration of the anti-osteoclast activity of APE on bone destruction, suggesting the potential promise of APE as an agent against bone-related disorders, including osteoporosis.

## Supplementary Materials

Supplementary materials can be accessed at: http://www.mdpi.com/1420-3049/19/8/11628/s1.

## References

[1] Feng X., McDonald J.M. (2011). Disorders of bone remodeling. Annu. Rev. Pathol..

[2] Raisz L.G. (2005). Pathogenesis of osteoporosis: concepts, conflicts, and prospects. J. Clin. Investig..

[3] Christenson E.S., Jiang X., Kagan R., Schnatz P. (2012). Osteoporosis management in post-menopausal women. Minerva Ginecol..

[4] Boyce B.F., Xing L. (2007). Biology of RANK, RANKL, and osteoprotegerin. Arthritis Res. Ther..

[5] Takayanagi H. (2007). Osteoimmunology: Shared mechanisms and crosstalk between the immune and bone systems. Nat. Rev. Immunol..

[6] Wada T., Nakashima T., Hiroshi N., Penninger J.M. (2006). RANKL-RANK signaling in osteoclastogenesis and bone disease. Trends. Mol. Med..

[7] Mizukami J., Takaesu G., Akatsuka H., Sakurai H., Ninomiya-Tsuji J., Matsumoto K., Sakurai N. (2002). Receptor activator of NF-kappaB ligand (RANKL) activates TAK1 mitogen-activated protein kinase kinase kinase through a signaling complex containing RANK, TAB2, and TRAF6. Mol. Cell. Biol..

[8] Gingery A., Bradley E., Shaw A., Oursler M.J. (2003). Phosphatidylinositol 3-kinase coordinately activates the MEK/ERK and AKT/NFkappaB pathways to maintain osteoclast survival. J. Cell Biochem..

[9] Mao D., Epple H., Uthgenannt B., Novack D.V., Faccio R. (2006). PLCgamma2 regulates osteoclastogenesis via its interaction with ITAM proteins and GAB2. J. Clin. Invest..

[10] Takayanagi H., Kim S., Koga T., Nishina H., Isshiki M., Yoshida H., Saiura A., Isobe M., Yokochi T., Inoue J. (2002). Induction and activation of the transcription factor NFATc1 (NFAT2) integrate RANKL signaling in terminal differentiation of osteoclasts. Dev. Cell.

[11] Matsumoto M., Kogawa M., Wada S., Takayanagi H., Tsujimoto M., Katayama S., Hisatake K., Nogi Y. (2004). Essential role of p38 mitogen-activated protein kinase in cathepsin K gene expression during osteoclastogenesis through association of NFATc1 and PU.1. J. Biol. Chem..

[12] Yagi M., Miyamoto T., Sawatani Y., Iwamoto K., Hosogane N., Fujita N., Morita K., Ninomiya K., Suzuki T., Miyamoto K. (2005). DC-STAMP is essential for cell-cell fusion in osteoclasts and foreign body giant cells. J. Exp. Med..

[13] Lee S.H., Rho J., Jeong D., Sul J.Y., Kim T., Kim N., Kang J.S., Miyamoto T., Suda T., Lee S.K. (2006). v-ATPase V0 subunit d2-deficient mice exhibit impaired osteoclast fusion and increased bone formation. Nat. Med..

[14] Song I., Kim J.H., Kim K., Jin H.M., Youn B.U., Kim N. (2009). Regulatory mechanism of NFATc1 in RANKL-induced osteoclast activation. FEBS Lett..

[15] Miyamoto H., Suzuki T., Miyauchi Y., Iwasaki R., Kobayashi T., Sato Y., Miyamoto K., Hoshi H., Hashimoto K., Yoshida S. (2012). Osteoclast stimulatory transmembrane protein and dendritic cell-specific transmembrane protein cooperatively modulate cell-cell fusion to form osteoclasts and foreign body giant cells. J. Bone Miner. Res..

[16] Arai A., Mizoguchi T., Harada S., Kobayashi Y., Nakamichi Y., Yasuda H., Penninger J.M., Yamada K., Udagawa N., Takahashi N. (2012). Fos plays an essential role in the upregulation of RANK expression in osteoclast precursors within the bone microenvironment. J. Cell Sci..

[17] Ha H., Shim K.S., Kim T., An H., Lee C.J., Lee K.J., Ma J.Y. (2014). Water extract of Acer tegmentosum reduces bone destruction by inhibiting osteoclast differentiation and function. Molecules.

[18] Luo K.W., Ko C.H., Yue G.G., Lee J.K., Li K.K., Lee M., Li G., Fung K.P., Leung P.C., Lau C.B. (2014). Green tea (Camellia sinensis) extract inhibits both the metastasis and osteolytic components of mammary cancer 4T1 lesions in mice. J. Nutr. Biochem..

[19] Xu R.S., Zong X.H., Li X.G. (2009). Controlled clinical trials of therapeutic effects of Chinese herbs promoting blood circulation and removing blood stasis. Zhongguo Gu Shang.

[20] Jia N., Li Y., Wu Y., Xi M., Hur G., Zhang X., Cui J., Sun W., Wen A. (2012). Comparison of the anti-inflammatory and analgesic effects of Gentiana macrophylla Pall. and Gentiana straminea Maxim., and identification of their active constituents. J. Ethnopharmacol..

[21] Hausmann E., Raisz L.G., Miller W.A. (1970). Endotoxin: stimulation of bone resorption in tissue culture. Science.

[22] Orcel P., Feuga M., Bielakoff J., De Vernejoul M.C. (1993). Local bone injections of LPS and M-CSF increase bone resorption by different pathways in vivo in rats. Am. J. Physiol..

[23] Ikeda T., Kasai M., Utsuyama M., Hirokawa K. (2001). Determination of three isoforms of the receptor activator of nuclear factor-[kappa]B ligand and their differential expression in bone and thymus. Endocrinology.

[24] Khosla S. (2001). Minireview: the OPG/RANKL/RANK system. Endocrinology.

[25] Hofbauer L.C., Gori F., Riggs B.L., Lacey D.L., Dunstan C.R., Spelsberg T.C., Khosla S. (1999). Stimulation of osteoprotegerin ligand and inhibition of osteoprotegerin production by glucocorticoids in human osteoblastic lineage cells: Potential paracrine mechanisms of glucocorticoids-induced osteoporosis. Endocrinology.

[26] Findlay D.M., Atkins G.J. (2011). Relationship between serum RANKL and RANKL in bone. Osteoporosis Int..

[27] Boyle W.J., Simonet W.S., Lacey D.L. (2003). Osteoclast differentiation and activation. Nature.

[28] Suda T., Kobayashi K., Jimi E., Udagawa N., Takahashi N. (2001). The molecular basis of osteoclast differentiation and activation. Novartis Found. Symp..

[29] Hayman A.R. (2008). Tartrate-resistant acid phosphatase (TRAP) and the osteoclast/immune cell dichotomy. Autoimmunity.

[30] Teitelbaum S.L. (2000). Bone resorption by osteoclasts. Science.

[31] Luxenburg C., Geblinger D., Klein E., Anderson K., Hanein D., Geiger B., Addadi L. (2007). The architecture of the adhesive apparatus of cultured osteoclasts: From podosome formation to sealing zone assembly. PLoS One.

[32] Stenbeck G. (2002). Formation and function of the ruffled border in osteoclasts. Semin. Cell Dev. Biol..

[33] Vaananen H.K., Horton M. (1995). The osteoclast clear zone is a specialized cell-extracellular matrix adhesion structure. J. Cell Sci..

[34] Nakamura I., Pilkington M.F., Lakkakorpi P.T., Lipfert L., Sims S.M., Dixon S.J., Rodan G.A., Duong L.T. (1999). Role of alpha(v)beta(3) integrin in osteoclast migration and formation of the sealing zone. J. Cell Sci..

[35] Mulari M.T., Zhao H., Lakkakorpi P.T., Vaananen H.K. (2003). Osteoclast ruffled border has distinct subdomains for secretion and degraded matrix uptake. Traffic.

[36] Schlesinger P.H., Blair H.C., Teitelbaum S.L., Edwards J.C. (1997). Characterization of the osteoclast ruffled border chloride channel and its role in bone resorption. J. Biol. Chem..

[37] Wagner E.F., Matsuo K. (2003). Signalling in osteoclasts and the role of Fos/AP1 proteins. Ann. Rheum. Dis..

[38] Macian F., Lopez-Rodriguez C., Rao A. (2001). Partners in transcription: NFAT and AP-1. Oncogene.

[39] Cremasco V., Decker C.E., Stumpo D., Blackshear P.J., Nakayama K.I., Nakayama K., Lupu T.S., Graham D.B., Novack D.V., Faccio R. (2012). Protein kinase C-delta deficiency perturbs bone homeostasis by selective uncoupling of cathepsin K secretion and ruffled border formation in osteoclasts. J. Bone Miner. Res..

[40] Stroup G.B., Lark M.W., Veber D.F., Bhattacharyya A., Blake S., Dare L.C., Erhard K.F., Hoffman S.J., James I.E., Marquis R.W. (2001). Potent and selective inhibition of human cathepsin K leads to inhibition of bone resorption *in vivo* in a nonhuman primate. J. Bone Miner. Res..

