# Supplementary material for: Aconitum pseudo-laeve var. erectum Inhibits Receptor Activator of Nuclear Factor Kappa-B Ligand-Induced Osteoclastogenesis via the c-Fos/nuclear Factor of Activated T-Cells, Cytoplasmic 1 Signaling Pathway and Prevents Lipopolysaccharide-Induced Bone Loss in Mice
Source: Molecules. 2014 Aug 5;19(8):11628–44. doi: 10.3390/molecules190811628 (PMC6270969; doi:10.3390/molecules190811628)

Supplementary Information

Figure S1. The effect of APE on RANKL and OPG expression in primary osteoblasts. Primary osteoblasts were cultured in 10% α-MEM with or without IL-1 (10 ng/mL) in the presence or absence of APE (200 μg/mL). Total RNA was isolated from cells using QIAzol reagent and mRNA expression levels of RANKL and OPG were evaluated by real-time RT-PCR.

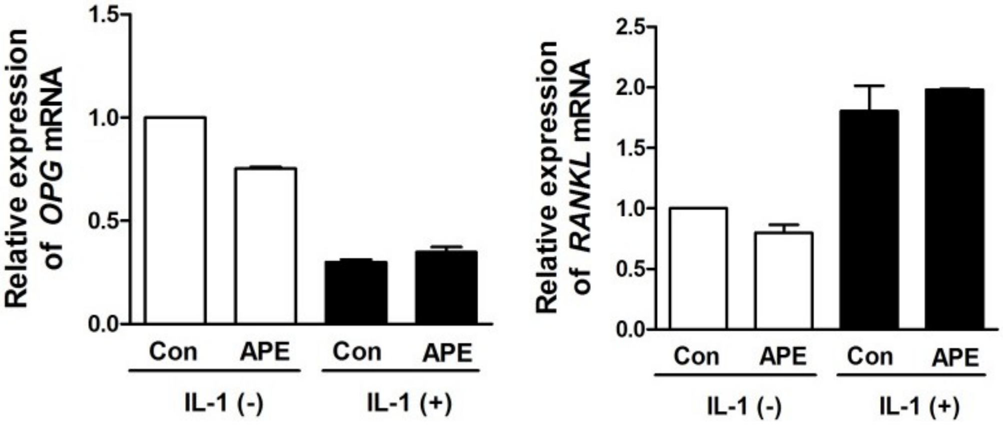

Figure S2. BMMs were infected with retroviruses expressing pMX-IRES-EGFP, pMX-c-Fos-EGFP, and pMX-CA-NFATc1-EGFP. Infected BMMs were cultured for 12 h or 48 h, and western blot analysis was performed for c-Fos or NFATc1 expression levels, respectively.

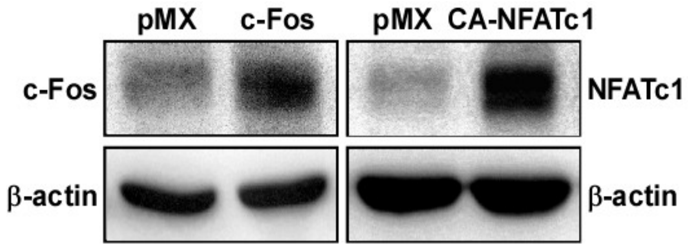

Supplement: Supplementary file 1 [file molecules-19-11628-s001.pdf]
